# Design of RNA splicing analysis null models for *post hoc* filtering of Drosophila head RNA-Seq data with the splicing analysis kit (Spanki)

**DOI:** 10.1186/1471-2105-14-320

**Published:** 2013-11-09

**Authors:** David Sturgill, John H Malone, Xia Sun, Harold E Smith, Leonard Rabinow, Marie-Laure Samson, Brian Oliver

**Affiliations:** 1 National Institute of Diabetes and Digestive and Kidney Diseases, National Institutes of Health, 50 South Drive, Bethesda, MD 20892, USA; 2 Program in Computational Biology, Bioinformatics, and Genomics, University of Maryland, College Park, MD 20742, USA; 3 Department of Molecular and Cell Biology, University of Connecticut, Storrs, Connecticut 06269, USA; 4 CNRS UMR 8195, Centre de Neurosciences Paris-Sud, Univ Paris-Sud, Orsay F-91405, CEDEX, France

## Abstract

**Background:**

The production of multiple transcript isoforms from one gene is a major source of transcriptome complexity. RNA-Seq experiments, in which transcripts are converted to cDNA and sequenced, allow the resolution and quantification of alternative transcript isoforms. However, methods to analyze splicing are underdeveloped and errors resulting in incorrect splicing calls occur in every experiment.

**Results:**

We used RNA-Seq data to develop sequencing and aligner error models. By applying these error models to known input from simulations, we found that errors result from false alignment to minor splice motifs and antisense stands, shifted junction positions, paralog joining, and repeat induced gaps. By using a series of quantitative and qualitative filters, we eliminated diagnosed errors in the simulation, and applied this to RNA-Seq data from *Drosophila melanogaster* heads. We used high-confidence junction detections to specifically interrogate local splicing differences between transcripts. This method out-performed commonly used RNA-seq methods to identify known alternative splicing events in the *Drosophila* sex determination pathway. We describe a flexible software package to perform these tasks called Splicing Analysis Kit (Spanki), available at http://www.cbcb.umd.edu/software/spanki.

**Conclusions:**

Splice-junction centric analysis of RNA-Seq data provides advantages in specificity for detection of alternative splicing. Our software provides tools to better understand error profiles in RNA-Seq data and improve inference from this new technology. The splice-junction centric approach that this software enables will provide more accurate estimates of differentially regulated splicing than current tools.

## Background

Alternative splicing generates different RNA molecules from identical primary transcripts, affecting protein diversity by creating diverse mRNA isoforms and modulating regulatory information in non-coding and untranslated regions in mRNAs [1]. The advance of next-generation sequencing technologies has allowed the high-throughput analysis of whole transcriptomes by RNA-Seq. In a typical RNA-Seq experiment, Poly-A^+^ transcripts are enriched from a pool of RNA, from which cDNA is generated, amplified, and sequenced [2]. Analysis of RNA-Seq data entails inferring the transcript molecule corresponding to each read, along with estimation of relative abundances of transcribed and processed features [2,3]. Thus, RNA-Seq experiments have the potential to produce novel discoveries and facilitate tremendous progress on understanding mRNA diversity generated by splicing.

Despite the promise, there are important sources of ambiguity, bias, and noise in RNA-Seq data that have made accurate estimation of splicing differences difficult in practice. These problems arise at multiple steps in an RNA-Seq experiment. At the library preparation stage, sequence-dependent variation in amplification generates heterogeneous coverage artifacts [4,5] that lead to differences in exon read counts even in constitutively spliced genes. At the sequencing stage, cluster generation allows sequencing of only a portion of the library, leading to sampling biases and variation between technical replicates [6]. At the alignment stage, reads with sequencing errors derived from paralogs and low sequence complexity regions confound abundance differences due to the preference for alignability over gap introduction [7]. These problems have complicated the analysis of splicing by RNA-Seq. While performing simulations of RNA-Seq data generation is a common approach to benchmarking tool performance and characterizing errors, and several tools exist that perform simulations (BEERS [8], maq (Heng Li, http://maq.sourceforge.net/), Flux Simulator [9], and ART [10]), these tools do not provide reporting that can easily be used to understand how aligner error affects downstream inferences on splicing, limiting utility.

Current strategies for quantifying splicing differences from RNA-Seq data employ isoform abundance estimations (Cuffdiff [11]), exon counts (DEXSeq [12]), and counts to pre-defined local regions (MISO [13]). Intron-centric splicing quantification has been proposed [14], and splice junctions alone have been shown to accurately quantify alternative splicing in cassette exons [15]. In addition to this variety of measurements, there are multiple units of comparison used to identify splicing differences. Classification of splicing differences between isoforms is non-trivial for complex gene models, and incomplete identification of these differences leads to ascertainment bias.

We developed a suite of tools called the Splicing Analysis Kit (Spanki) to model, analyze, and improve junction detection, and to enable a complete splice-junction centric analysis of RNA-Seq data (Table 1). This software is available at http://www.cbcb.umd.edu/software/spanki and https://github.com/dsturg/Spanki.

**Table 1:** Comparison of features among RNA-Seq analysis tools

***Feature***	**Spanki**	**Tophat**	**Cufflinks**	**MISO**	**DEXSeq**	**RUM**	**Flux capacitor**	**Maq**
Simulation tools	x					x	x	x
Empirical error modeling	x						x	x
Custom simulated transcript coverages	x							
Junction alignment curation	x	x^1^						
Gene assignment for junctions	x		x^2^					
Qualitative junction analysis	x							
Junction-level comparisons	x							
Event-level comparisons	x		x^3^	x	x^4^			
PSI metric reporting	x			x				

^1^Tophat offers criteria for filtering what is reported after the alignment stage. Spanki provides additional criteria that can be applied after reporting.

^2^Cufflinks assembles transcripts and merges with annotated genes.

^3^Cuffdiff reports differential splicing by TSS group, without specifiying the differential splicing event.

^4^DEXSeq provides results for exon-level abundance differences.

Spanki analyzes and mitigates error profiles, based on simulations that closely mimic real data. Uniquely, the Spanki read simulator combines robust empirical modeling with detailed reporting that is geared toward evaluating splicing detection performance. This allows the production of simulations that approximate real experimental error profiles; and that, which can be applied to help develop an analysis pipeline or to generate a custom error profile for every sample. Our modeling based on real RNA-Seq sequencing errors, coupled with simulations, reveals multiple sources of false positive junctions. Spanki calculates and reports junction alignment diagnostics for *post hoc* alignment filtering methods to ensure accurate junction quantification.

We show that splice junctions provide a more direct and less ambiguous measurement of splicing than exon read counts of full length isoform abundance measurements. To address the problem of splicing event classification, we apply standardized and exhaustive splicing event ontologies with AStalavista [16] and show that mutually exclusive splicing differences are effectively interrogated using junctions. The Spanki software therefore demonstrates a complete set of routines for splice-junction centric analysis of RNA-Seq data.

As a test case, we examined splicing in *Drosophila melanogaster* female and male heads. We chose these samples for two reasons. First, the central nervous system of many species is highly complex in architecture and is a rich source of alternative transcripts [17]. Additionally, the *Drosophila* sex determination hierarchy is a classical model of regulated alternative splicing [18]. Three members of this hierarchy, *Sex-lethal* (*Sxl), transformer* (*tra*), and *male specific lethal 2* (*msl-2*) encode broadly expressed alternatively spliced mRNAs. The two terminal members of the hierarchy *doublesex* (*dsx*) and *fruitless* (*fru*) are also alternatively spliced and are expressed in a restricted set of neurons, in addition to other non-neuronal tissues. We demonstrate that our approach produces alternative splicing measurements that are consistent with the literature and quantitative PCR (qPCR) results, and provides superior detection of sex-differential splicing than other methods. In benchmarking tests with a null dataset, we show a lower false positive rate for differential splicing calls than commonly used tools and a moderate false negative rate.

## Results and discussion

### Analysis overview

Analysis of alternative splicing with RNA-Seq data involves multiple interdependent components including mapping reads, identifying pairwise splicing differences, and quantifying alternative splicing. A variety of tools perform individual tasks, and null models are critical tools for evaluating how well these tools perform. We built a suite of tools called the Splicing Analysis Kit (Spanki) to generate null models from simulations, evaluate aligner performance, and quantify splicing differences. This toolkit is modular in design and can be used as a complete analysis pipeline, to evaluate exisiting pipelines, or to make informed decisions on parameters (Table 1).

We used these methods to show that reads that directly detect intron removal (junction spanning reads) provide a basis for a complete analysis of splicing with advantages in specificity and low type I and type II error rates. We demonstrated these advantages with simulated datasets and real biological data.

### Approach to error modeling

**Figure 1:**
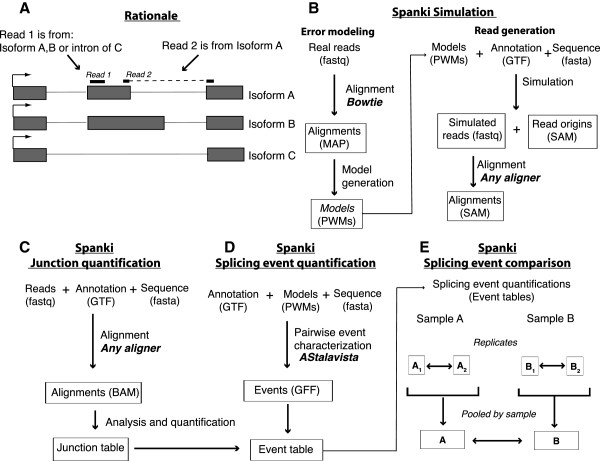
**Rationale and overview of analysis approach. (A)** Cartoon of a hypothetical locus encoding alternatively spliced transcripts, illustrating how junction spanning reads map unambiguously to specific introns. Read 1 could have originated from the 2^nd^ exon of isoform **A** or **B**, or the intron of isoform **C**; while read 2 could only have originated from isoform A and the indicated splice junction. **(B-E)** Flowcharts of analysis steps performed in Spanki. Input data listed at the top, format in parentheses, and calls to external programs indicated (bold). **(B)** Flowchart of simulation methods. A two step process begins with modeling error profiles based on a permissive Bowtie [19] alignment. These error models are used by the simulator to generate reads. **(C-E)** Flowcharts of quantification and comparison methods. The first step is junction quantification **(C)**, where alignments are performed, junction alignments are curated, and junction coverages are calculated. Splicing event quantification **(D)**, where a set of transcript models (from annotation or computed using a program such as Cufflinks [11]), are used to characterize pairwise splicing differences (“splicing events”). These events are merged with junction coverage data to quantify the mutually exclusive paths defined for each event. Splicing event comparison **(E)** uses these tabulated event-level quantifications to compare between replicates, and between pooled results for each sample, by Fisher’s Exact Test on inclusion and exclusion junction counts.

A common approach to examining splicing is to determine read coverage of alternative exons, assemble full length isoform models, and generate probabilistic abundance estimates of the alternative forms [7]. The inherent problem with this type of approach is that reads mapping to exon space may originate from multiple alternative exons with different exon boundaries (Figure 1A) [2]. Additionally, both read coverage heterogeneity and intron retention makes calling alternative splicing from exon counts problematic. In contrast, reads that span splice junctions derive from a true splicing event and unambiguously join exons, making this a much more useful measurement [8]. However, mapping these reads is more difficult than alignment to a contiguous genomic reference, making high quality junction alignments critical for downstream analyses [8]. Since junction detection is the foundation of our analysis, we undertook simulations to quantitatively assess splice junction detection performance so that we could characterize and then filter out dubious junctions. We built simulated datasets in two steps: modeling and read generation (Figure 1B).

The first step in the analysis of junction-based splicing detection is to identify and quantify the junction spanning reads (where part of the read aligns to one exon and another part to another exon Figure 1A). We performed this analysis using an annotation (Figure 1C) or without. We then merged junction coverage data and estimated the relative abundance of the alternative forms (Figure 1D). In the next step we classified splicing events from annotated transcript models to obtain sets of junctions that define mutually exclusive “paths” (Inclusion and Exclusion) that interrogate each path specifically. This allows us quantify alternative splicing using the *Percent Spliced In* (PSI) metric [20], which is simply the abundance of the inclusion form divided by the sum of the inclusion and exclusion forms. To find the number of genes alternatively spliced, we selected events for which junction coverage was detected over the inclusion path in either sample, and over the exclusion path in either sample and performed statistical testing (Figure 1E).

### Error models and simulations

**Figure 2:**
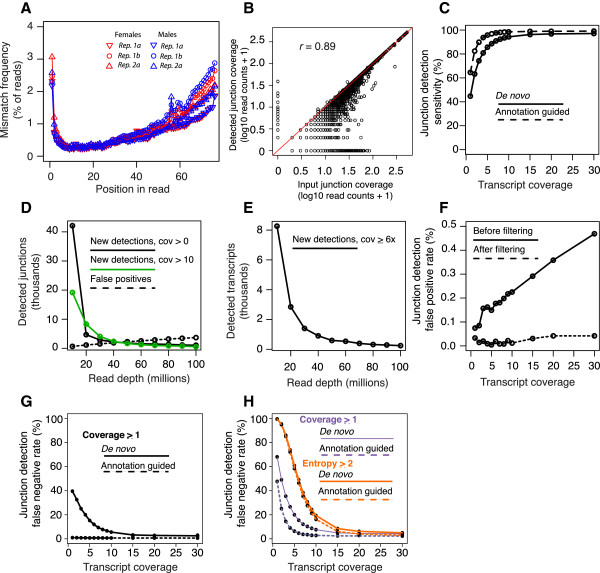
**Simulation results and junction detection. (A)** Actual mismatch frequency by read position. Replicates (technical and biological) of female (red) and male samples (blue) are indicated. **(B)** Accuracy of annotated junction detection. Recovered junction coverage (y-axis) compared to actual coverage (x-axis) in simulated input. Read counts (+1) in log10 scale. **(C)** Sensitivity of junction detection (1 – false negative rate). Receiver operator characteristic (ROC) curve of splice junction detection displays sensitivity as it relates to sequencing depth. Results represent TopHat mapping with annotation (dashed line), and without annotation (solid line). **(D)** Junction detection in subsamples of real data in read pools of increasing sequencing depth (10–100 million reads in increments of 10 million). Junctions detected with at least one read (black line), or with ≥10 reads (green line) are indicated. For each pool, the additional junctions detected relative to the previous pool are indicated. Total cumulative false positive junction detections in each pool (dashed line). **(E)** Transcript coverage in subsamples of real data. Annotated transcripts detected with at least 6x coverage (black line) in each subsampled pool of real data. **(F)** Junction detection false positive rate in simulated data pre- (solid line) and post-filtering (dashed line). False positive rate is in percent of all annotated junctions with simulated reads, and is not cumulative. **(G)** False negative rate of junction detection due to alignment failure (i.e., not due to sampling), when at least one junction spanning read is generated from simulated transcripts. **(H)** False negative rate of junction detection due to sampling. Rates are for detecting at least one junction spanning read (coverage ≥ 1, purple lines), or for detecting an entropy score ≥ 2 (orange lines).

We aligned RNA-Seq reads with permissive parameters (quality aware alignment, with no fixed mismatch cutoff) using Bowtie [19] in order to estimate total mismatch profiles along the full length of the reads. As has been previously reported, we observed increased mismatch rates extending through the 3′ end of the read and a slight increase in mismatch rates in the first 5 bases [4,21,22] (Figure 2A). We determined nucleotide mismatch frequencies by position in the read and by substitution type. These frequencies are used by the Spanki read simulator as weights in a weighted-random selection, to choose the total number of mismatches in a read, mismatch positions, and base substitution. We supplied these error models to the Spanki read simulator to generate a defined known input sample generated *in silico* from annotated transcript models, with error profiles matching these empirical models.

To generate simulated reads, we extracted transcript sequence from each *D. melanogaster* annotated gene model and generated 13 pools of simulated 76 bp paired-end reads at 1-30X coverage where the error profiles matched our real data. This produced pools where transcript coverage is equalized, allowing us to examine the coverage-dependent effects on detection. To model retained introns, due to either regulation or incomplete processing, we generated 20% of the reads from transcript models with introns included. We applied this elevated rate of intron retention (empirical estimate is 6.9 - 7.2%, unpublished) intentionally to increase aligner error. Modeled error frequencies were applied as weights for mismatch number, position, and substitution. To enable the tracking of aligner errors, we incorporated the genomic coordinates of origin for each read into a unique read identifier. We then uniquely aligned the reads using TopHat [23], and compared alignment results to the known input to explore splice site detection parameters. This two-step process generated a simulated data set that mirrors the experimental dataset, except that the true input was known, providing us a platform for testing RNA-Seq junction alignment. Results that follow provide evaluation results for the TopHat aligner [23], although the same approach can be applied to any aligner output.

We compared junction coverage with known input abundance for all junctions in the 10x transcript coverage pool (Figure 2B). Since multiple transcripts at a locus may share a given junction, individual junction coverage was 1-400x (median 8x, 4.2 million read pairs) reflecting both the random sampling of read positions and overlapping *D. melanogaster* transcript models at a given locus. Our junction coverage measurements had high concordance with simulated input (Pearson’s r = 0.89) demonstrating that junction coverage closely tracks known input.

Junction spanning reads are a small portion of the total reads in an RNA-Seq experiment (9.4-12.6% in the six samples used in this study) raising the possibility that sufficient coverage for calling junctions would be problematic. To test for the effects of read depth on the false negative rate, we generated pools of simulated reads for each annotated reference transcript at multiple fixed coverage levels (1-10x, 15x, 20x, and 30x) and aligned these simulated reads with a reference annotation ('Annotation guided’) or without ('*De novo*’), and compared detection results with known input (Figure 2C). We detected >90% of junctions with 3x simulated transcript coverage when we provided an annotation. Without the benefit of annotation, we found that 6x coverage was required to reach this level of sensitivity. Reaching this level of coverage for each annotated transcript (63 million bp of transcript sequence) required 2.5 million read pairs (5 million total reads). To put this in context of experimental data, we typically detect > 8,000 transcripts at ≥ 6x coverage with 5 million mapped reads in *Drosophila* RNA-Seq experiments.

We simulated sequencing depths by sampling in 10 million read increments from one high-depth experiment by random selection (without replacement), and evaluated the relationship of read depth, detection of junctions, and detection of annotated transcripts in each pool. This enables us to evaluate detection in which relative transcript abundances match the biological sample. In this analysis, we define “new” detections as features that are not detected in a lower-depth pool. We found that > 40,000 junctions (> 65%) were detected in the first 10 million reads and that a 10-fold greater read depth added ~20,000 more junctions (Figure 2D). At depths of > 50 million mapped reads, the number of cumulative false positive detections exceeded the cumulative number of new junction detections (Figure 2D), as well as the number of new junctions detected robustly (≥10 reads), and the number of new annotated transcripts detected with at least 6x coverage began to level off (Figure 2E). Additionally, the contribution to detected isoform complexity diminished with added depth, as new detections were increasingly from single exon and constitutively spliced genes. We did not observe over-representation of any splicing event type with increased depth. We generated normalized transcript abundance estimates in units of fragments per kilobase per million mapped reads (FPKM), and found that we obtained 6x coverage of 95% of the transcripts reliably detected at FPKM ≥ 1 in the full dataset (200 million mapped reads). We examined the false positive rate at multiple transcript coverage levels (Figure 2F) and found that the rate increased with greater transcript coverage due to cumulative errors in alignment. These data indicate that greater read depth provides more opportunities to call false positives. However, the majority of the false positives can be filtered *post hoc* (Figure 2F) as we explain later.

### False negative junction detection

Junction detection is a function of sampling within a sequenced transcript, aligner performance, and multiple isoforms sharing a junction (Figure 2C). To separate these factors, we analyzed junction detection false negative rates in constitutively spliced genes (where a junction is only present in one isoform).

We removed the effect of sampling by analyzing detection when at least one junction spanning read is generated in the simulation. This effectively gives us the false negative rate of generating an alignment when at least one junction spanning read is present (Figure 2G). When the aligner was provided with the annotation, the false negative rate of alignment was 1% at 1x transcript coverage. Without an annotation, the false negative rate was 40%, but declined to < 10% at 7x coverage. These results show that coverage requirements are modest when working with genomes with well-defined transcript models. Without an annotation, islands of read density are required to generate a reference of putative junctions; so false negative rates at low coverage are high. At high transcript coverage, the false negative rate of junction alignment was modest. We estimated the false negative rate of alignment using the detection deficit at 30x transcript coverage observed in Figure 2B. We divided total detected junction spanning reads by total simulated input, and obtained a false negative rate of 3.6%. We also examined junctions that differ in a small number of nucleotides from other junctions (≤10 bp apart). We found higher false negative rates for this class of junctions (6.6%). These overlapping junctions pose more difficulty for the aligner to detect, but they represent a small fraction of annotated donors (1.1%) and acceptors (1.6%).

When we removed the requirement that a junction spanning read was generated, we found false negative rates to be driven primarily by sampling (Figure 2H). False negative rates were 48%-68% at 1x transcript coverage. If we apply the entropy cutoff criterion, we find much higher false negative rates, since at least four unique alignment offsets are required to meet this entropy ≥ 2 threshold. False negative rates did not decline below 50% until 6x transcript coverage, illustrating that quantitative filtering is overly stringent for the detection of rare variants.

The qualitative criteria we described are not sequencing-depth dependent, and hence have no relationship to transcript coverage. One criterion (sequence repetitiveness) can be applied without an annotation, and we estimate the false negative rate of applying this criterion at the 80% threshold is 0.33% (180 annotated junctions have ≥ 80% repetitiveness).

### False positive junctions

RNA-Seq experiments can reveal splice junctions that are not yet annotated. Distinguishing novel detection from experimental error in this class of junctions is a major challenge. Even though the false positive rate was < 0.5%, with tens of thousands of junctions detected, even these low error rates generated hundreds of false positives that would be counted as novel splice junctions in experimental data sets. Junction detection errors have far-reaching downstream effects such as calls incorrectly supporting gene merges, antisense transcripts, and alternative splicing events.

**Figure 3:**
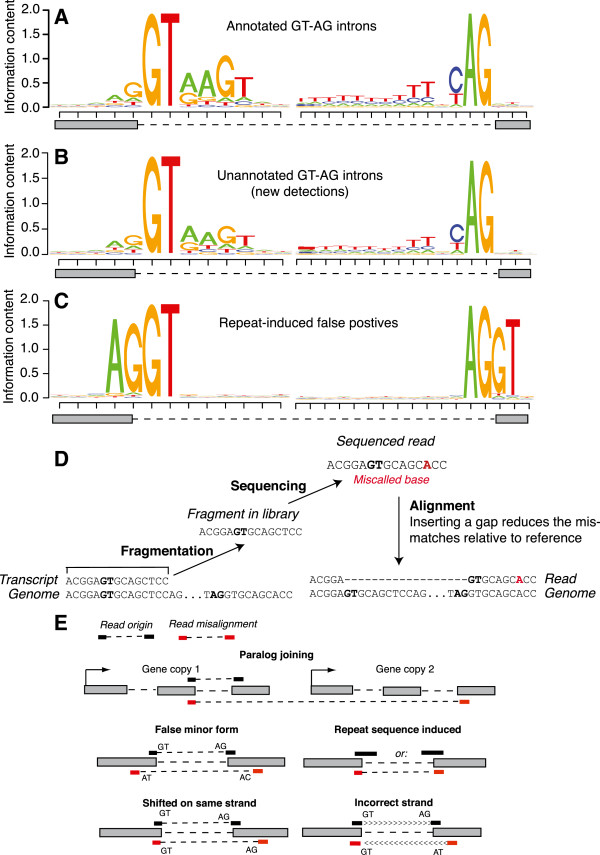
**Sequence characteristics of false positive junctions.** Sequence logos of exon and intron sequence bordering splice junctions in **(A)** annotated GT-AG introns, **(B)** unannotated GT-AG introns detected that pass filtering, and **(C)** repeat induced false positives. **(D)** Cartoon illustrating how false positives arise from repetitive sequence and sequencing error. A transcribed fragment from a region of repetitive sequence is incorporated into a library. A base calling error (in red) produces a read with an “A” instead of a “T” at the indicated position. This incorrect base call induces an incorrect gapped alignment that minimizes sequence mismatches. **(E)** Illustration of each false positive error type (Table 2).

While there are multiple sources of error in junction calling (Table 2), the dominant error source was due to the aligner introducing junctions to reduce mismatch rates. This can be illustrated by examining extended motifs in introns. The most common donor/acceptor motif pattern is GT-AG, and these major forms have additional well-defined motifs within the intron sequence whether they are from annotated or un-annotated junctions (Figure 3A, B). However, in low sequence complexity regions with either very high or very low % GC (>70% or <10%), mismatches induced a more optimal alignment when the read was split and joined to another segment up or downstream. This type of error can be clearly seen by the absence of an extended motif (branch sites and polypyrimidine tracts), and over representation of the motif “AGGT” on both ends of the junction (Figure 3C, D). We performed additional simulations consisting only of contiguous genomic sequence to estimate the frequency of this error class. 10 million simulated reads from contiguous genome sequence resulted in 310 false positive junctions. Thus, in an RNA-Seq experiment with, for example, contamination from genomic DNA, repeat-induced errors will be generated at a rate of 1 per 36,000 contaminating reads. Given that an intron is often used as evidence for a transcript and not contaminating DNA [24], these errors can lead to false calls of intergenic transcription when accompanied with artifactual coverage islands in intergenic space. Confidence in junction calls in intergenic space is therefore critical in resolving the existence of pervasive transcription [25].

**Table 2:** Sources of false positive junction detection

**Type of error**	**False positives**	**Qualitative filtering strategy**	**Removed by qualitative filtering**^**1**^	**Removed by quantitative filtering**^**2**^
False alignment to minor form	36.4%	Remove novel minor forms	36.4%	30.7%
Incorrect strand	31.6%	Inconsistency with gene model	31.6%	28.8%
Shifted on same strand	13.8%	None	0%	12.2%
Paralog joining	8.5%	Inconsistency with gene model	8.5%	7.7%
Repeat sequence induced	7.7%	Exon-intron sequence similarity	7.7%	6.5%
Unidentified error	2%	None	0%	0%
***Total defined errors:***	100%	***Total removed errors:***	84.2%	75.9%

^1^False positives removed by Spanki’s qualitative filtering.

^2^False positives removed by filtering on entropy score (≥ 2), calculated by Spanki.

To lower the false positive rate, it is important to understand the nature of the errors. We examined sources of alignment error leading to false positives at 30x coverage and classified them (Table 2, Figure 3E). One major source of error was false detection of junctions from rare “minor-form” (AT-AC and GC-AG) introns, which represent a small fraction of introns in *D. melanogaster* annotation [26], and less than 0.5% of introns across metazoan lineages [27]. Although AT-AC introns are > 100X rarer than GT-AG introns in the annotation (0.027% of junctions), TopHat chose the more optimal alignment, resulting in the false placement of a GT-AG spliced alignment on a proximal AT-AC site because of an alignment with fewer mismatches at a proximal AT-AC site than to the correct GT-AG site. Introns with the AT-AC dinucleotide are similarly rare in other species (0.10% of human introns, 0.09% of mouse introns, and 0.02% of Arabidopsis introns [26]). The preference for optimal alignment with fewer mismatches also led to false positive alignments on incorrect strands. In RNA-Seq data from non-strand-specific protocols, the strand is inferred from the sequence of the interior donor/acceptor motif. For example, a shift in the 3′ end of the alignment causes a (+) strand GT-AG intron to be read as a (-) strand minor form GT-AT intron. If uncorrected, errors of this type lead to the false prediction of antisense transcripts. Mismatches resulted in alignment to the wrong site in the gene model. Within this class of errors, 33% correctly place one end of the alignment (the donor or the acceptor), 12% of them incorrectly join annotated donors and acceptors from different transcripts of the same gene, and the remainder place neither donor or acceptor correctly. These pernicious errors result in the false appearance of alternative isoforms. Similarly, the joining of paralog exons, which reside proximally in the genome, occurred when a splice junction originating from one paralog was aligned as a join between separate paralogous genes, falsely merging distinct genes into a single model. This class of error may be more prominent with aligners that allow indels or gene fusions. For example, we found paralog joining in 24% of false positives called by TopHat2 [28].

### Filters

After characterizing error sources, we sought to remove as many as reasonably achievable (Table 2). We first examined the effectiveness of a simple quantitative cutoff on the alignment Shannon’s entropy score [29], a metric that quantifies alignment complexity based on diversity of alignment offsets. Requiring an entropy score ≥ 2 for each junction removed 75.9% of false positives. However, since quantitative filtering criteria are overly stringent in the case of rare transcripts, we developed a series of qualitative criteria that removed 84.2% of false positives while allowing analysis of low abundance junctions.

To prevent strand switches and gene merges at paralogs, we identified the most likely gene of origin of each donor and acceptor based on genomic overlap and strand, and required agreement. Junctions were flagged as “ambiguous” if each edge was assigned to a different gene or if either end was assigned to no gene, allowing us to filter them out. We found that filtering on this simple criterion was effective in removing all false positive junctions in simulated data where a junction was called on the wrong strand or if paralogs were incorrectly joined (40.1% of false positives, Table 2).

To filter repeat sequence induced errors, we used the edit distance between exon shoulder sequence and intron sequence. For each junction, Spanki compared 10 bp upstream of the donor to 10 bp upstream of the acceptor, and 10 bp downstream of the donor to 10 bp downstream of the acceptor, and reported the percent identity. Using a threshold of 80%, this comparison revealed cases where similarity between putative exon and intron sequence generated false gapped alignments. We found that filtering junctions where introns were > 80% identical to up or downstream exon sequence removed all these errors (7.7% of false positives, Table 2). To remove cases where mismatches induced alignment to a minor form intron, we removed introns of this minor class when they were not annotated (36.4% of false positives, Table 2).

Applying the qualitative filtering criteria above removed 84.2% of false positive junctions in our simulated data. The remaining 15.8% of false positives were qualitatively identical to true positives and could not be filtered. These false positives are consistent with the strand of the gene model and are adjacent to canonical donor and acceptor dinucleotides. While we do not evaluate them here, machine learning methods that evaluate extended sequence motifs [30] hold promise for filtering these errors. Nevertheless, qualitative criteria removed 8.4% more false positives than using entropy scores alone. Importantly, we achieved this reduction in false positives without requiring junctions to be detected with high coverage. Our abundance independent qualitative filtering led to an overall false positive rate of < 0.04% across all simulated read depths.

Junction filtering is critical for accurately defining the splicing event landscape of the transcriptome, as each false positive can incorrectly define alternative donors, acceptors, and cassettes. Studies in organisms with incompletely annotated genomes rely heavily on empirically detected junctions. Spanki’s design allows the flexible application of these filters, which is critical to accommodate different sample types and alignment strategies. For example, aberrant splicing may be a feature of interest in mutant or cancer samples, rather than an artifact to filter out [31]. Although we present results using the first generation TopHat aligner, other tools allow searching for fusion transcripts (TopHat2, [28]), or non-canonical splicing variants (MapSplice, [32]). In these cases, simulation allows for assessment of error rates and selective application of filters.

### Differential splicing detection

To identify cases of differential splicing between samples, it is essential to find where splicing patterns diverge to define a unit of comparison. Basic categories of alternative splicing incompletely describe complex splicing patterns, which can lead to under-reporting of differences. We applied standardized and exhaustive splicing event ontologies with AStalavista [16] to ensure that pairwise splicing differences are interrogated completely. Spanki parses AStalavista output to obtain sets of junctions that define mutually exclusive “paths” (Inclusion and Exclusion), to identify junctions that interrogate each path specifically. We use the detection of coverage over these junctions to calculate PSI.

**Figure 4:**
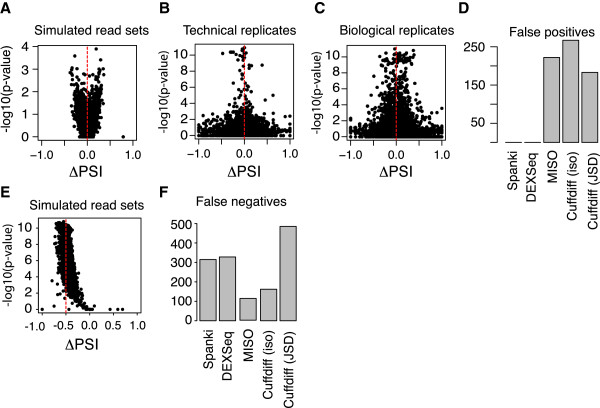
**Variation in Percent Spliced-In (PSI) and error rates.** Volcano plots where ΔPSI is plotted against the –log10 p-value of the Fisher’s Exact Test, to assay variation due to sampling and sequencing error in simulations **(A)**, technical RNA-Seq replicates **(B)**, and biological replicates **(C)**. **(A)** Plot comparing two simulated read sets of equal reads per kilobase (300 Reads Per Kilobase). Since transcript abundances are equal, expected ΔPSI is zero. **(B)** Variation in ΔPSI between replicate RNA-Seq runs of the same libraries. **(C)** Variation in ΔPSI between independent biological samples, each with a distinct RNA-Seq library. Results within each sex were similar, results for female samples shown. **(D)** False positive differential splicing calls in a null dataset. Cufflinks results are shown for both splicing analysis (Jensen-Shannon Divergence, JSD), and isoform abundance comparison. MISO results shown are based on isoform-centric analysis. **(E)** Volcano plot of ΔPSI calculated by Spanki for false negative analysis, comparing simulated datasets with PSI = 0.25 and PSI = 0.75 respectively, for 1644 events (expected ΔPSI = -0.50, red line). **(F)** Counts of false negatives for differential splicing calls in simulated data with an input ΔPSI = -0.50.

To analyze false positive calls of differential splicing, we generated a Spanki null model for splicing differences by simulating four read pools, each of which contained reads from all annotated transcripts in equal abundances of 300 Reads Per Kilobase (RPK). We then analyzed this null dataset, applying junction filtering and Spanki junction-based event definitions, and also compared technical and biological replicates of real RNA-Seq data to each other. We performed a systematic categorization of all pairwise relationships using AStalavista [16], which constructs graphs from transcript models and outputs complete and non-redundant sets of splicing differences identified through graph alignment (see Methods). We used these pairwise definitions to make comparisons on the PSI metric calculated by Spanki. We found minor variation due to sampling alone and technical replication (Figure 4A, B), but biological replication was a much greater source of variability (Figure 4C). Low total abundance events showed the most disagreement between replicates (< 10 average coverage per site in either path).

Next we compared the number of differential splicing calls made in our simulated null dataset by Spanki and by several other methods. Spanki correctly called zero events differentially spliced in this dataset (Figure 4D). We counted reads that map within exons using the script provided with DEXSeq [12], and performed an exon-level differential analysis. DEXSeq also called zero exons differentially expressed, however, with a reduced sensitivity to real alternative events in other data sets (not shown). Next we performed an isoform-centric analysis using MISO [13], which called differential splicing in transcripts of 222 genes. Analysis with Cuffdiff [11], with default parameters except for specifying upper quartile normalization, called 183 loci as differentially spliced, and 267 isoforms were called differentially expressed in our null input dataset with no true differences in splicing.

To analyze the false negative rate of differential splicing detection, we generated two simulated datasets with a known PSI splicing difference. To prevent cross-talk, we selected splicing events where both the inclusion and exclusion forms were composed of transcripts that were not part of any other splicing event (N = 1644 events), and simulated one pool so that PSI = 0.25 for all events, and a second pool with 0.75 PSI for all events, so that comparing the two would yield -0.50ΔPSI. After processing these data with Spanki, the ΔPSI values clustered at -0.50ΔPSI (median -0.46) (Figure 4E). Spanki failed to call significant differences in 315 events (19%) with FDR correction (Figure 4F). Cufflinks with the Jensen-Shannon Divergence (JSD) metric had a 29% false negative rate, but performed better when comparing isoform abundances, failing to find differential isoform expression in only 5% of isoforms where an abundance difference was simulated. At the exon level, DEXSeq had a false negative rate of 19%, but with false positive differences in 116 genes. MISO performed the best at this task, which did not detect a splicing difference in 115 genes (7% false negative rate). These results show that this simulation was a challenge for these tools and produced high false negative rates, but Spanki performed comparably to other tools at the same task.

This junction-based calculation of splicing differences is a more balanced and less biased measure than exon counts. Although exon counts yield more data, ambiguity of assignment (Figure 1A), coverage heterogeneity [4], and unprocessed transcripts [33] make these data unreliable. Exon counts are also a more imbalanced measurement of alternative forms. For example, in the case of skipped exons, the cassette inclusion form can be interrogated by reads within the cassette, but the exclusion form has zero exonic space that can be uniquely interrogated. This imbalance can be extreme, as in the case of multiple large coordinate cassette exons. This means that exon counts provide an inaccurate measurement, and to only one side of a comparison of proportions, further compounding the bias.

### Splicing detection in *D. melanogaster* heads

**Figure 5:**
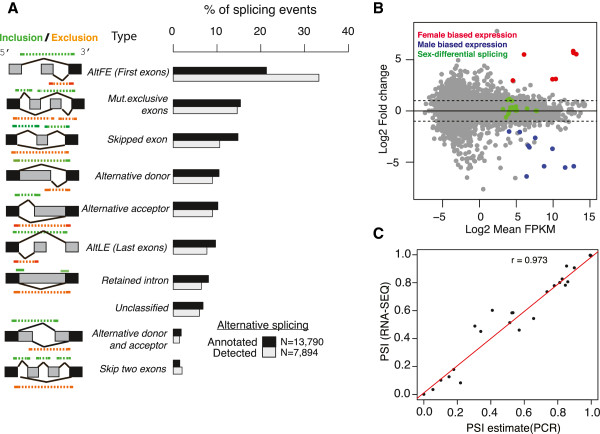
**Splicing event characterization in *Drosophila *heads. (A)** Pairwise splicing events defined for all transcript models in Flybase 5.39 annotation (black bars), and the subset of those detected as alternatively spliced in female and male *Drosophila* heads by Spanki (grey bars). Black bars indicate splicing event classes as a percent of all defined pairwise events. Grey bars indicate splicing events detected as a percent of all detected events. Cartoons of each event type are in the leftmost column, with the “inclusion” form indicated in green, and the “exclusion” form indicated in orange. A description of each type is adjacent to each cartoon. The “Unclassified” types includes diverse complex type with no concise verbal description. **(B)** Ratio vs average abundance scatterplots of gene expression in female and male heads. Genes with significant sex-biased gene-level expression (FDR adjusted p < 0.05, Cuffdiff) in females (red), or males (blue). Remaining detected expression events are in grey. Transcripts showing sex-biased splicing have been projected onto the gene-level expression data (green). **(C)** Scatter plot of percent spliced in (PSI) by RNA-Seq vs qPCR for the inclusion or exclusion form for each event assayed (the PCR primer pairs differ for detecting exon inclusion or exclusion but approximates PSI). Inset is Pearson’s r coefficient.

To test the performance of *post hoc* filtering and alternative splicing detection, we generated RNA-Seq data on sexed *D. melanogaster* heads (Additional file 1: Table S1). Spanki quantified 70,827 filtered junctions arising from 5,329 genes in our *D. melanogaster* head RNA-Seq data (Additional file 2: Table S2). To analyze the full repertoire of splicing complexity, we analyzed all pairwise relationships defined by AStalavista [16]. This analysis yielded 13,790 pairwise-defined alternative splicing events (Figure 5A). Of these, 9,201 were internal events (not involving the first or last exons) and the remaining 4,589 were alternative promoter events. While alternative promoter use is not alternative splicing *per se,* we included these in our analysis since isoforms from alternative promoters are biologically relevant. Our method discriminates alternative first exons that differ in their splicing, and excludes overlapping first exons that differ only in their five-prime ends. The majority of splicing events were cassette exons, mutually exclusive exons, alternative donors, alternative acceptors, alternative first and last exons, and retained introns. However, 1,306 internal events (14%) did not fit into the seven basic categories. Of this class, “Skip two exons” (200 events) was the largest category, followed by “Alternative donor and acceptor” (two variants, 142 and 137 events, respectively). This latter category is an example of a structure overlooked by classifications into basic categories [1]. An additional 827 events (125 unique structures) are termed “Unclassified” because they have no concise verbal description, but these do receive a code describing the graph pattern. The top five occurring structures in the “Unclassified” category comprise 41.5% of these events, each of which represent a variant of a skipped exon event.

We then used Spanki to merge junction coverage data and estimate the relative abundance of the alternative forms. We found that 7,894 splicing events in transcripts from 2,441 genes were alternatively spliced in head samples (5,450 internal events in 1,852 genes) (Figure 5B). We found 182 events with significant differences between female and male heads (adjusted p-value < 0.01, Benjamini and Hochberg, Additional file 3: Table S3). To conservatively adjust the sex-biased expression calls for rare events and biological variability as outlined previously, we demanded >10 junction reads in each path, and that the unadjusted p-value for the between**-**sexes comparison was less than the unadjusted p-value between biological replicates. In light of the variance we observed in our null dataset (Figure 4A) and between biological replicates (Figure 4C), we also set a conservative threshold on the difference between samples at > 0.20 PSI. This filtering yielded 22 events in 17 genes significantly different between the sexes (Table 3), including members of the sex-determination cascade. Strikingly, the genes showing sex-biased transcription profiles at the gene level are not the same genes that show sex-biased splicing (Figure 5B) suggesting that targets for sexually dimorphic expression are regulated by transcriptional or post-transcriptional mechanisms, but less so by both. Like sex-biased splicing, we find that sex-biased gene expression is modest in heads (19 genes), compared with the large amount of differential expression in the whole adults with gonad tissue [34]. These splicing events were validated by quantiative PCR in independent biological samples (Figure 5C). We found stronger agreement when either the inclusion or exclusion forms were dominant, than when proportions were more equal (PSI ~ 0.5, Figure 5C). This suggests that much more extensive biological replication is needed to resolve any regulated small differences in splicing from biological noise.

**Table 3:** Genes sex-differentially spliced

**Gene ID**	**Gene name**	**Chromosome**	**Event type**	**ΔPSI**^**1**^	**Adj. p-value**^**2**^	**GO annotation**^**3**^
FBgn0004652	*fru*	3R	altdonor	-1	5.87E-08	Male courtship behavior
FBgn0003659	*Sxl*	X	exonskip	0.974	5.87E-08	Sex determination
FBgn0000504	*dsx*	3R	AltLE	0.939	5.87E-08	Sex determination, male courtship behavior
FBgn0004652	*fru*	3R	exonskip	-0.906	9.90E-08	Male courtship behavior
FBgn0028341	*l(1)G0232*	X	AltFE	0.802	2.98E-08	Protein tyrosine phosphatase activity
FBgn0086675	*fne*	X	altdonor	-0.656	6.76E-08	Regulation of RNA metabolism
FBgn0005616	*msl-2*	2L	retintron	0.565	1.38E-03	Dosage compensation
FBgn0259923	*Sep4*	X	AltFE	-0.524	4.79E-04	GTPase activity
FBgn0259923	*Sep4*	X	altdonor	-0.469	5.87E-08	
FBgn0053113	*Rtnl1*	2L	AltFE	-0.464	6.39E-08	Inter-male aggressive behavior, olfactory behavior
FBgn0053113	*Rtnl1*	2L	AltFE	-0.444	5.87E-08	
FBgn0053113	*Rtnl1*	2L	AltFE	0.426	5.87E-08	
FBgn0004852	*Ac76E*	3L	exonskip	-0.382	5.87E-08	Intracellular signal transduction
FBgn0086674	*Tango13*	X	altdonor	0.372	6.61E-08	Sulfotransferase activity
FBgn0003741	*tra*	3L	altacceptor	-0.371	5.87E-08	Sex determination, male courtship behavior
FBgn0260660	*mp*	3L	skip2exons	-0.252	7.93E-03	Motor axon guidance
FBgn0259682	CG42351	2R	exonskip	-0.242	9.21E-08	*none*
FBgn0259214	*PMCA*	4	mutexcl	0.232	5.87E-08	Calcium transporting ATPase activity
FBgn0259214	*PMCA*	4	exonskip	-0.229	5.87E-08	
FBgn0037297	CG1116	3R	retintron	0.229	1.39E-03	*none*
FBgn0010482	*l(2)01289*	2R	Un-classified	0.22	2.98E-08	Protein disulfide isomerase activity
FBgn0036194	CG11652	3L	AltFE	0.208	8.26E-03	Phagocytosis

^1^PSI in females – PSI in males. Table is sorted by ΔPSI absolute value.

^2^p-value from Fisher’s Exact Test, FDR corrected by Benjamini-Hochberg.

^3^Selected terms (not a complete list), extracted from the “Summary information” section of each gene’s FlyBase entry [35].

Many of the sex-biased splicing events we observed may be important for sexual behavior based on known gene functions. *Reticulon-like 1* had significant differences at several pairwise defined alternative first exons. *Rtnl1* encodes a membrane protein localized to the endoplasmic reticulum [36] and has a role in inter-male aggressive behavior [37], olfactory response [38], and motor axon development [39]. Another gene with sex-differential skipped exons, *multiplexin*, is involved in motor axon guidance [40], although without a known link to behavior. We detected sex-differential regulation in transcripts encoded by the *found in neurons* (*fne*) gene, which encodes a member of the embryonic- lethal abnormal vision (ELAV) gene family of RNA-binding proteins [41,42]. Wildtype *fne* is required for robust male courtship behavior [43].

**Figure 6:**
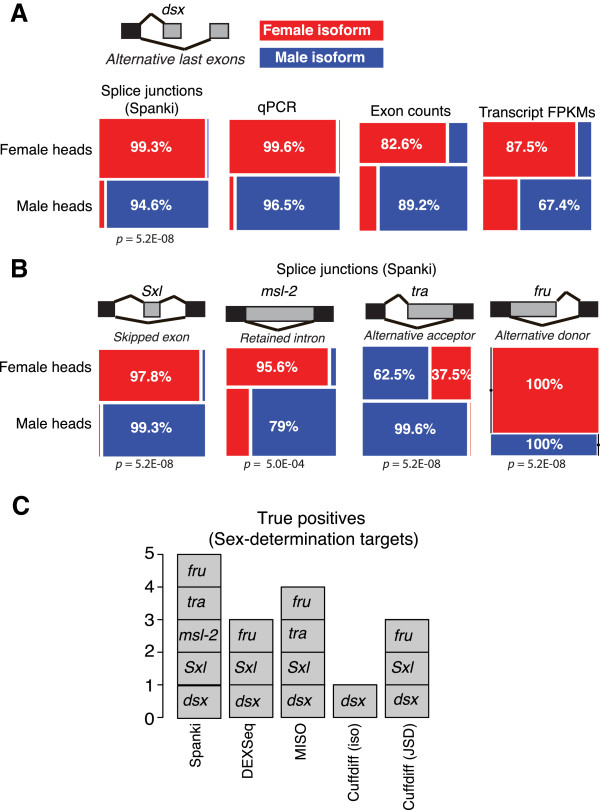
**Resolution of splicing differences in the sex determination pathway.** Detection and visualization of sex-differential splicing in sex determination pathway components by different methods. **(A)** Analysis of the regulated alternative last exons splicing event in *dsx*, showing the splicing difference between the female isoform (top, red) and male isoform (bottom, blue). Mosaic plots display female isoform (red) and the male isoform (blue) abundances in each sex for: splice junctions counts, qPCR, counts of reads within exons, and full length isoform abundance estimates (FPKM). **(B)** Splicing analysis for other components of the sex determination pathway, along with sex-differential splicing results obtained: *Sxl*, skipped exon; *msl-2*, retained intron, *tra*, alternative acceptor, and *fru*, alternative donor. Significance measures from Spanki (Benjamini-Hochberg adjusted p-value) are shown beneath each mosaic plot. **(C)** Performance of other RNA-Seq analysis tools in detecting sex determination pathway components. DEXSeq [12], which relies only on exon-level counts, detected significant differences in *fru*, *Sxl*, and *dsx*, but not in *msl-2* or *tra*. Similarly, a Bayesian analysis with MISO [13] failed to detect differential splicing of *msl-2* transcripts. For Cuffdiff [11], we examined results for the splicing difference test (Jensen-Shannon Divergence metric), and also for isoform abundance differences.

Our splicing calls for the sex determination transcripts were more sex-biased than in previous RNA-Seq experiments [29] on whole adult flies. To help determine if this was due to methodology, we also quantified splicing events using measurements of exon coverage and normalized isoform abundance estimates (in FPKM), to see if these approaches yielded similar results. These metrics predicted results that were much less sex-specific; for example in the case of *dsx* sex- specificity was 62.9- 67.4% by exon counts or FPKM, and 95.6-99.4% by Spanki (Figure 6A). There are possible technical explanations for the non-sex-specific exon read results, including the sequencing of transcripts that are polyadenylated before splicing is complete (more prevalent on the 3-prime end in *dsx*). Inefficient poly-A selection due to oligo (dT) priming in unprocessed transcripts may also play a role [33]. Our measurements using splice junctions better reflect processed transcriptional output. These results show that using junction coverage with Spanki results in more switch-like splicing difference calls. Similarly, we found highly sex-biased splicing of other members of the sex determination cascade, except for the *tra* locus where incomplete splicing to the female form is known to occur [44] (Figure 6B). None of the other splice detection methods called sex-biased splicing for each of the sex determination gene transcripts (Figure 6C), clearly highlighting the improvements made with filtered Spanki output.

As expected for any expression study based on sampling, we did see greater variability at low abundance, but when abundance was high, results were stable. At high simulation coverage, PSI quantification was also accurate (Figure 4E). The results for *dsx* and the other sex determination targets greatly exceeded the 10 junction counts threshold. Although higher counts do indeed give more stable measures of proportions, the effect we are observing in this case is sex-specificity, for which junction counts are a superior measurement.

## Conclusions

Junction-based splice calling is an important method for analysis of alternative splicing. Our results highlight many of the junction-read errors that can occur in these RNA-Seq datasets and outline simulation strategies for modeling these errors, either while developing an analysis pipeline, or tailored for each experiment. We have implemented tuneable filters in Spanki to remove false positives without sacrificing specificity, and clearly show that developing error models from real RNA-Seq data and applying these *post hoc* filters improves splicing detection in *D. melanogaster* heads. Failing to filter for sequencer and aligner error, to account for mapping ambiguity in transcriptome space, and to under-estimate the null distribution of splicing differences, results in inaccurate estimation of splicing differences in RNA-Seq studies.

## Methods

### Molecular biology

RNA samples were prepared from *white*^*1118*^*, Canton-S (B)* isogenic stock adult *D. melanogaster* heads [45,46]. 7 days post-eclosion flies were grown at low density, allowed to mate *ad libitum*, flash frozen on dry ice, and beheaded in biological duplicates. Sample descriptions and detailed methods are provided in Gene Expression Omnibus (GEO) accessions (GSM928376, GSM928377, GSM928383, GSM928384, GSM928392, and GSM928393). We added exogenous controls (1% final) from the External RNA Control Consortium (ERCC, pool 15) prior to library construction [4]. Paired-end sequencing was performed on GAII or HiSeq instruments (Illumina, San Diego, CA, USA) for 76 cycles for each read mate.

For quantitative real-time RT-PCR, 1 μg of total RNA was subjected to DNase treatment (Promega, Madison, WI, USA) followed by reverse transcription, using the random primer of the Transcriptor First Strand cDNA Synthesis Kit (Roche Applied Science, Indianapolis, IN, USA). PCR was performed in biological duplicates, with duplicate quantification of each biological duplicate. cDNA from 12.5 ng of total RNA was amplified with Fast SYBR Green Master Mix (Applied Biosystems, Carlsbad, CA, USA) in a StepOne Real-Time PCR machine (Applied Biosystems, Carlsbad, CA, USA). Initial activation was performed at 95°C for 20 seconds followed by 40 cycles. Cycles were 95°C for 3 seconds followed by 60°C for 30 seconds. Then the melting curve was generated ranging from 60°C to 95°C with an increment of 0.5°C each 5 seconds. *Act5c* (*Actin 5C*) was used as a control. Primers were designed with the web interface of the NCBI Primer-Blast software [47]. All amplification products were analyzed by agarose gel electrophoresis and produced single fragments of predicted sizes. The relative transcript level was calculated using the cycle threshold value (Ct) by the method of 2^-ΔCt^, where ΔCt = Ct_transcript_ - Ct_*Act5c*_. qPCR data are provided for each primer pair, after normalization to junction coverage of the mutually exclusive isoform.

### Read mapping

We used reads that passed Chastity (score ≥ 0.6) base-calling filtering (Illumina CASAVA pipeline 1.6.47.1) and mapped using TopHat v1.4.1 [11], with Bowtie v0.12.7 [19], and samtools 0.1.12a [48], and parameters “-g 1 –solexa1.3-quals, -i 42.” We used *D. melanogaster* genome release 5 [49,50], as obtained from the UCSC genome browser (excluding “chrUextra”) [51], for mapping. We also appended sequence for 96 exogenous controls to the genomic reference [4]. A reference annotation (Ensembl release 67, corresponding to Flybase 5.39) was also supplied in GTF format with the -G option. We made a minor modification in the annotation to remove the antisense transcripts of *modifier of mdg4* (FBgn0002781), since these transcripts caused fatal errors in downstream analysis tools.

### Gene expression quantification and comparison

To produce estimates of gene and transcript level abundance, we quantified based on both full-length transcript assemblies and on discrete counts within annotated genomic boundaries, as each approach has different strengths [11,52]. We used Cufflinks [11] (v.2.0.2) to generate abundance estimates of full-length isoforms, expressed in units of expected fragments per kilobase of transcript per million mapped reads (FPKM). We determined relative abundance differences using Cuffdiff v.2.0.2 [11], using upper quartile normalization, and setting “max-bundle-frags” very high (50E06), to ensure that very highly expressed features were not excluded. To provide alternative quantifications and comparisons, we used HTSeq [52] to generate counts of reads that fall within discrete features. The “htseq-count” program in HTseq v.0.5.3, with the conservative “union” mode and default parameters, was used to generate counts. We used the R package DESeq (v.1.8.3) to test for differential expression [52]. “Variance outliers” were identified as contrasts where the maximum residual variance is > 15. This value was exceeded in ~2% of all genes, which we removed from our final differential expression calls.

### Simulation and splicing analysis

Simulations of junction reads, along with quantification of junctions and alternative splicing, was performed in the open-source python package: Splicing Analysis Kit (Spanki). A summary of Spanki’s features is provided in Table 1. Spanki is available at http://www.cbcb.umd.edu/software/spanki and http://github.com/dsturg/Spanki.

Error models were built by performing quality-aware mapping on our reads with Bowtie v0.12.7 [19], and supplying the map file to the program *spankisim_models*. These models are incorporated into the Spanki repository, so that they can be applied to simulations by choosing the “*flyheads*” error model.

For generating simulated reads, we used the *spankisim_transcripts* command, supplying a reference annotation (Ensembl release 67, corresponding to Flybase 5.39), using the default parameters for intron retention (-ir = 0.20) and fragment size (-frag = 200 bp). The coverage to simulate was specified as either coverage values with the –cov parameter (for the simulations in Figure 2), or as reads per kilobase with the –rpk (for the simulations in Figure 4). We used the “*flyheads*” error model, which was built as described above, and is included in the program. The simulated reads in this study are available here: http://www.cbcb.umd.edu/software/spanki/simulations.html.

We also compared our detection performance to other tools, including Cuffdiff, MISO, and DEXSeq. We used the results reported in the “splicing.diff” file from Cuffdiff v.2.0.2 [11]. We used MISO with event definitions for *D. melanogaster* from the MISO website, supplemented with additional custom definitions, and applied a Bayes factor cutoff > 40 for the false positive analysis, and > 10 for the false negative analysis [13]. For simulated null datasets, we performed isoform centric analysis using Ensembl annotation. For DEXSeq, we counted reads that map within exons using the script provided with the package [12], and performed an exon-level differential analysis.

#### Spanki program design (Simulator)

Spanki estimates error model parameters from a first pass alignment of real RNA-Seq reads using permissive quality aware mapping with Bowtie [19]. The error modeling function within Spanki parses the alignments in Bowtie’s *map* format, and produces probability weight matrices for mismatches by position in the read and by base substitution type, and for quality scores by position. The read simulator uses these models to introduce mismatches.

Spanki’s RNA-Seq simulator function generates simulated reads with errors incorporated. The user sets the transcripts to simulate (e.g. transcripts expressed in the biological sample under study), a depth of coverage (e.g. matching the experimental sequence depth), and mismatches are then introduced according to the specified model. If a user does not want to build new error models, pre-built error models from *D. melanogaster* head RNA-Seq described in this work are included, along with models based on a sample from the modENCODE developmental timecourse [29], and a simple weighted-random model. The Spanki simulator takes transcript models in GTF format, and extracts transcript sequence from a genomic reference and chooses random positions in the designated transcript sequence to extract reads. For paired-end reads, fragment sizes are drawn from a normal distribution of mean 200 bp and standard deviation 20 bp. This is user tunable. For example, to simulate intron retention, Spanki generates a specified fraction of simulated reads from complete transcript sequence where introns are retained. Depth of coverage is specified in units of transcript coverage or reads-per-kilobase (RPK). Reads can be generated for transcripts in fixed proportions, creating a null model for splicing differences between samples. Alternatively, Spanki accepts a text file where the user can list individual transcripts to simulate at different coverages, which allows simulating fixed quantitative splicing differences between alternative isoforms, or the user can specify a custom model built on the user’s own data.

Modeled error frequencies are applied as weights for mismatch number, position, and substitution. Weight matrices of quality scores are used to create a consensus quality value across all positions - one for matched positions, and one for mismatched positions, which are concatenated to create a quality string for the read. Spanki reports information that facilitates analysis of alignment and detection. Coverage generated by the simulation for each splice junction is reported, along with read counts for each transcript. To enable the tracking of aligner errors, the genomic coordinates of origin for each read are incorporated into a unique read identifier. The true origin of simulated reads is also reported in a SAM file that represents a perfect alignment, which can be fed to an assembler such as Cufflinks [11] to allow the evaluation of error in transcript abundance estimates due to assembly separately from errors in alignment.

#### Spanki program design (Junction filtering quantification)

For maximum flexibility, Spanki decouples the alignment and filtering steps, with a tool that applies post-hoc analyses of alignment files. This allows alignments to be performed on multiple data sets, with consistent filtering applied later, and allows changing the filtering criteria without re-aligning. Spanki streams through a BAM file produced by any aligner, using the Pysam module (Andreas Hager, http://code.google.com/p/pysam/) and calculates junction coverage along with alignment diagnostic measurements. These measurements include the number of alignment offsets, alignment entropy [29], and Minimum Match on Either Side (MMES) [53]. Qualitative diagnostic results are also reported, such as repetitiveness of exon anchor and intronic sequence. Two values are calculated, the edit distance of 5-prime exon sequence and 3-prime intron sequence, and the edit distance of 3-prime exon sequence and 5-prime intron sequence. This operation is performed on 10 bp segments, but the user can specify other sizes. Gene assignments for junctions are reported to identify possible paralog joining errors. Gene assignments are made for each donor and acceptor site by genomic overlap with annotated gene models, and the consensus of both is used as the junction gene assignment. When the gene assignments for each end of a junction do not agree, they are reported as ambiguous.

In addition to alignment diagnostic values, Spanki generates calculations that are informative of splicing regulation. For example, Spanki estimates intron retention for each junction, regardless of the presence of an annotated retained intron isoform, by quantifying “intron read-through.” These are read alignments that span the exon/intron boundary without gaps on either side. To ensure comparability, Spanki enforces an overhang requirement, which is user-tunable, and is applied to both intron read-through and junction calling.

#### Spanki program design (Splicing event definition and quantification)

While there are only a few major splicing type events, the full diversity of possible splice forms is much more complex. Spanki provides utilities for parsing essentially any splicing event using definitions produced by AStalavista [16]. The AStalavista algorithm begins by decomposing transcript models into “sites,” which are exon boundaries. Graphs are built for each gene, where splice sites are nodes and intron or exon edges connect them. Splicing events are subgraphs with identical nodes on ends, but no common interior nodes. This process finds regions of the parent transcript where the donor/acceptor sites of two alternatives are present on a parent transcript, but utilized mutually exclusively in processed transcripts. Spanki uses these event definitions to build mutually exclusive “paths” composed of disjoint junction sets that interrogate each event specifically.

Uniquely, Spanki reports coverage from joins to exons that are outside of the event being considered. This is because many gene models are complex, and splicing events cannot always be assayed independently. For events with multiple exons in the inclusion or exclusion paths, there may be up- and down-stream connections to other exons that confound results. To adjust for this, Spanki calculates and reports the junction coverage for first-order neighbors of all interior exons that extend to exons outside the local splicing event. This coverage may lead to over-or under-counting of inclusion or exclusion joins within the splicing event. Since our model focuses on discrete and specific measurements, we use this information to indicate the presence of potentially confounding coverage for each event.

Since splicing analysis is a comparison of two alternative events, it is convenient to compare using proportions. The PSI metric that Spanki uses to express proportions has been applied elsewhere to splicing microarrays and RNA-Seq [20,54,55]. Using only junctions yields more consistent comparisons between events than including exon reads, since the number of positions is constant for events of the same type. Since different splicing paths may be composed of a different number of junctions (for example, in the case of skipped exons), the PSI metric is calculated as the number of reads per junction in the inclusion path divided by the number of reads per junction in the inclusion path plus the reads per junction in the exclusion path.

Assessing the significance of differences between samples requires accounting for differences in transcription and sequencing depth. The Fisher’s Exact Test (FET) is well suited to this task, since testing proportions accounts for differences in sample totals due to depth or transcription. Spanki constructs 2 × 2 contingency matrices from junction counts for each splicing event, to test the null hypothesis that the two samples have equal inclusion/exclusion proportions. The two cells of the first row of the matrix are the total read counts of the inclusion and exclusion junctions, respectively, for one sample. The second row contains the same data for the second sample. The test as constructed uses integer counts, as required for the Fisher’s exact test. Each defined pairwise event is tested, and each gene may have multiple events. The test is performed using the fisher python package v.0.1.4 (Brent Pederson, http://pypi.python.org/pypi/fisher/). FDR correction is performed by the Benjamini-Hochberg method implemented in the StatsModels package (Skipper Seabold, Josef Perktold, http://statsmodels.sourceforge.net/).

To help visualize splicing differences, Spanki includes R scripts to produce mosaic plots, where the relative size of each cell is proportional to real (non-normalized) cell counts. Code is also included to produce fourfold plots, which provide a visual test of the null hypothesis of the FET. This provides an effective simultaneous visualization of normalized proportions and significance. These plots are implemented in the “vcd” package for R [56].

## Competing interests

The authors declare that the have no competing interests.

## Authors’ contributions

DS, MLS, LR and BO designed the study. JHM, XS, HES, and MLS generated data. DS and MLS performed the analysis. DS and BO designed wrote the manuscript. All authors read and approved the final manuscript.

## Supplementary Material

Additional file 1 RNA-Seq read depth. Click here for file

Additional file 2 Details of junction detection in Drosophila heads. Click here for file

Additional file 3 Sex-differential results in wild-type heads. Click here for file
